# Case Report: Adebrelimab-associated suspected immune-related myocarditis with myositis/myasthenia gravis overlap syndrome (IM3OS): A rapidly fatal case and clinical implications

**DOI:** 10.3389/fonc.2026.1778682

**Published:** 2026-06-19

**Authors:** Hongyan Ji, Haijun Qu, Donghua Liu, Wen Xu, Xinyi Wang, Mengna Cui, Qie Guo, Fanbo Jin, Yuanxin Miao

**Affiliations:** 1 Department of Pharmacy, The Affiliated Hospital of Qingdao University, Qingdao, Shandong, China; 2 Department of Plastic Surgery, The Affiliated Hospital of Qingdao University, Qingdao, Shandong, China

**Keywords:** adebrelimab, IM3OS, immune checkpoint inhibitors, immune-related adverse events, myasthenia gravis, myocarditis

## Abstract

This article reports a case of a 72-year-old male with intrahepatic cholangiocarcinoma who developed acute symptoms—including chest tightness, muscle weakness, ophthalmoplegia, and dyspnea—3 days after receiving the second cycle of adebrelimab, following combination therapy with adebrelimab, apatinib, and tegafur. These complications proved fatal. Laboratory tests showed significant elevation of myocardial injury markers (high-sensitivity troponin T, N-terminal pro-B-type natriuretic peptide, myoglobin, and creatine kinase). Electrocardiogram showed multi-lead ST-T changes and conduction abnormalities, and neurological examination showed ophthalmoplegia. Immune checkpoint inhibitor-associated myocarditis with myositis/myasthenia gravis overlap syndrome (IM3OS) was highly suspected. Despite immediate drug discontinuation and progressive immunosuppression with corticosteroids and intravenous immune globulin, the patient’s condition worsened, and cardiac arrest occurred on day 9. This case underscores the rapid onset and poor prognosis associated with adebrelimab-induced suspected IM3OS, highlighting the critical need for rigorous baseline assessment, prompt recognition, early initiation of aggressive immunosuppressive therapy, and coordinated multidisciplinary management to prevent or mitigate these potentially life-threatening immune-related adverse events.

## 1 Introduction

The application of immune checkpoint inhibitors (ICIs) has notably improved the survival prognosis of patients with various advanced malignancies, representing a significant milestone in cancer immunotherapy. Currently, it is extensively utilized in the treatment of small-cell lung cancer, triple-negative breast cancer, esophageal squamous cell carcinoma, and other malignant tumors. Nevertheless, while activating the immune system to target tumors, ICIs may also lead to a series of immune-related adverse events (irAEs), which can affect multiple organ systems, including the skin, gastrointestinal tract, endocrine glands, liver, and lungs (1). Among these, ICI-related myocarditis is one of the most severe irAEs, with an incidence of approximately 1% but a high mortality rate (2). Complicating matters further, myocarditis can co-occur with other irAEs, such as myositis or myasthenia gravis, forming what is known as “immune-related myocarditis with myositis/myasthenia gravis overlap syndrome” (IM3OS).

Immune checkpoint inhibitor-associated IM3OS is a rare but highly fatal immune-related adverse event, with an in-hospital mortality rate ranging from 38% to 60%. This syndrome typically manifests early during immunotherapy, with a median onset of 21 days after the first dose, and the majority of patients develop symptoms after receiving only one treatment cycle. Epidemiologically, it is more common in elderly patients (median age 70 years), with a male predominance (3). The clinical manifestations are characterized by acute and progressive features, typically presenting with the onset of nervous system symptoms or involvement of the cardiovascular system. Neurological symptoms encompass proximal limb weakness, neck weakness accompanied by difficulty in raising the head, as well as indicative oculomotor symptoms (bilateral ptosis, diplopia, and limited eye movement) and bulbar symptoms (dysphagia, dysarthria). The cardiovascular system is manifested by chest tightness, dyspnea, orthopnea, tachycardia, and low-pitched and muffled heart sounds. Laboratory tests reveal a pattern of significant elevation: a marked increase in creatine kinase and a substantial elevation of troponin (often tens of times the upper limit of normal), frequently accompanied by elevated transaminases and myoglobin. Electrocardiography most commonly shows conduction system abnormalities (atrioventricular block), and cardiac magnetic resonance can demonstrate myocardial edema and late gadolinium enhancement. The disease progresses rapidly, and the primary causes of death are respiratory failure and malignant arrhythmia (3, 4).

Its pathophysiological mechanism remains incompletely understood. However, it is hypothesized that it is primarily associated with the excessive activation of T cells induced by immune checkpoint inhibitors subsequent to immune suppression. The heart, skeletal muscle, and neuromuscular junction may possess common antigenic targets. Through molecular mimicry mechanisms, activated CD8+ cytotoxic T lymphocytes can infiltrate these tissues concurrently, leading to myocarditis, myositis, and neuromuscular junction injury. Additionally, the blockade of the PD-1/PD- L1 signaling axis disrupts peripheral immune tolerance. Some patients exhibit elevated levels of autoantibodies (e.g., anti-acetylcholine receptor (AChR) antibodies and anti-titin antibodies) prior to treatment, along with abnormal activation of the complement system, which may contribute to the onset and progression of this syndrome (5–7).

Currently, clinical reports of IM3OS induced by PDL1 inhibitors, particularly adebrelimab, are extremely scarce, and its specific clinical characteristics, risk factors, and optimal management strategies still require further exploration.

This study presents a case of suspected IM3OS in an elderly patient diagnosed with intrahepatic cholangiocarcinoma, who was treated with the adebrelimab, apatinib, and tegafur regimen. Through a detailed presentation of its clinical progression, diagnostic difficulties, and treatment predicaments, this case intends to enhance clinicians’ comprehension of this catastrophic, multi-system immunotoxicity and emphasize the utmost significance of early recognition, risk alert, and multidisciplinary collaborative intervention in the era of potent combination therapy.

## 2 Case presentation

The patient was a 72-year-old man weighing 75 kg. He was admitted to the hospital on June 29, 2025, due to general weakness accompanied by dyspnea for 3 days, with the symptoms worsening in the past 1 day. He had a history of hypertension for over 20 years and a 7-year history of type 2 diabetes. He had been taking amlodipine, metoprolol succinate, acarbose, aspirin enteric-coated tablets, and atorvastatin for a long period. He had a smoking history of more than 20 years and occasional drinking habits. He quit smoking and drinking 2 years ago.

**Figure 1:**
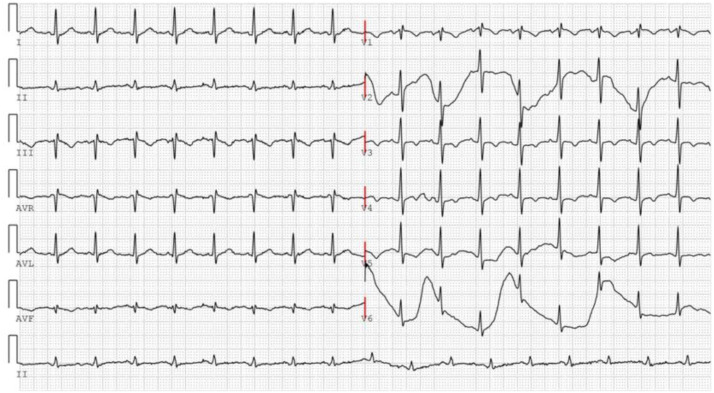
Electrocardiogram at the patient’s initial presentation on May 14.

Prior to the current admission, the patient was hospitalized on May 14, 2025, for poorly differentiated adenocarcinoma of the intrahepatic bile ducts. Laboratory evaluation at that time revealed direct bilirubin (DBil) of 8.82 μmol/L, gamma-glutamyl transferase (GGT) of 243 U/L, alkaline phosphatase (ALP) of 147 U/L, and lactate dehydrogenase (LDH) of 272 U/L. Cardiac biomarkers showed high-sensitivity cardiac troponin T (hs-cTnT) <0.003 μg/L (within normal limits), N-terminal pro-B-type natriuretic peptide (NT-proBNP) of 487.9 pg/mL, and myoglobin of 127.5 μg/L. Electrocardiography demonstrated sinus tachycardia with ST-T wave abnormalities in multiple leads (**Figure 1**). Coronary CT angiography revealed three-vessel coronary artery disease with multiple luminal stenoses, including a focal segment with findings suggestive of moderate to severe stenosis in the left circumflex artery. An ultrasound - guided needle biopsy of liver nodules showed that the pathological diagnosis was poorly differentiated adenocarcinoma, and immunohistochemistry results were CK19 (+) and CK7 (+). On May 22, 2025, the patient successfully underwent transarterial chemoembolization (TACE). During the procedure, raltitrexed (2 mg) and oxaliplatin (100 mg) were infused, followed by embolization with pirarubicin (30 mg) mixed in lipiodol (10 mL). The patient was discharged on May 24 without any post-procedural symptoms.

On June 5, 2025, the patient initiated systemic antitumor therapy, which consisted of adebrelimab (1200 mg, administered intravenously every 3 weeks), oral apatinib (250 mg, once daily), and tegafur (40 mg, twice daily). On June 26, 2025, the patient revisited the hospital as scheduled and successfully concluded the second cycle of adebrelimab infusion.

**Figure 2:**
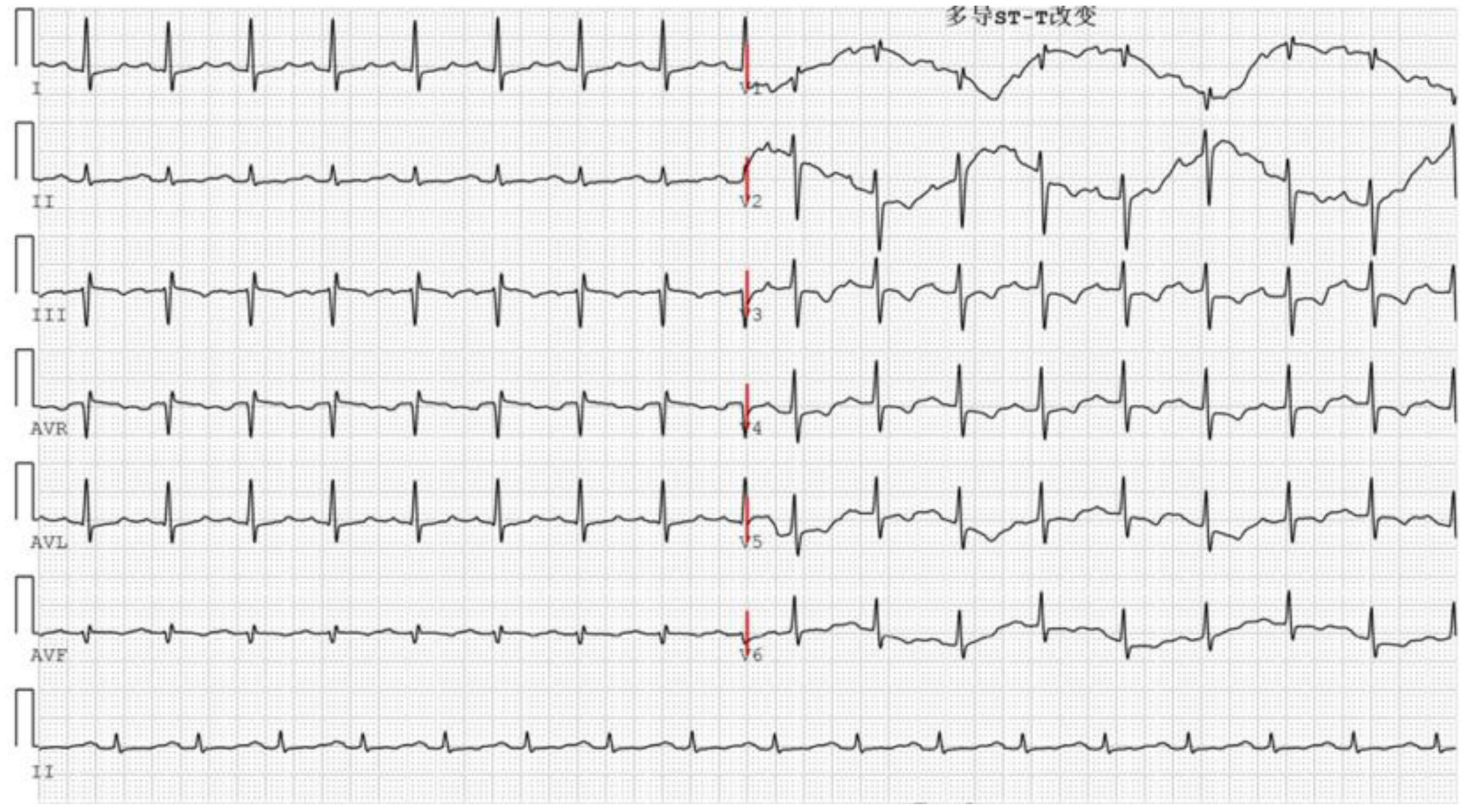
The patient’s ECG at admission on June 29.

**Figure 3:**
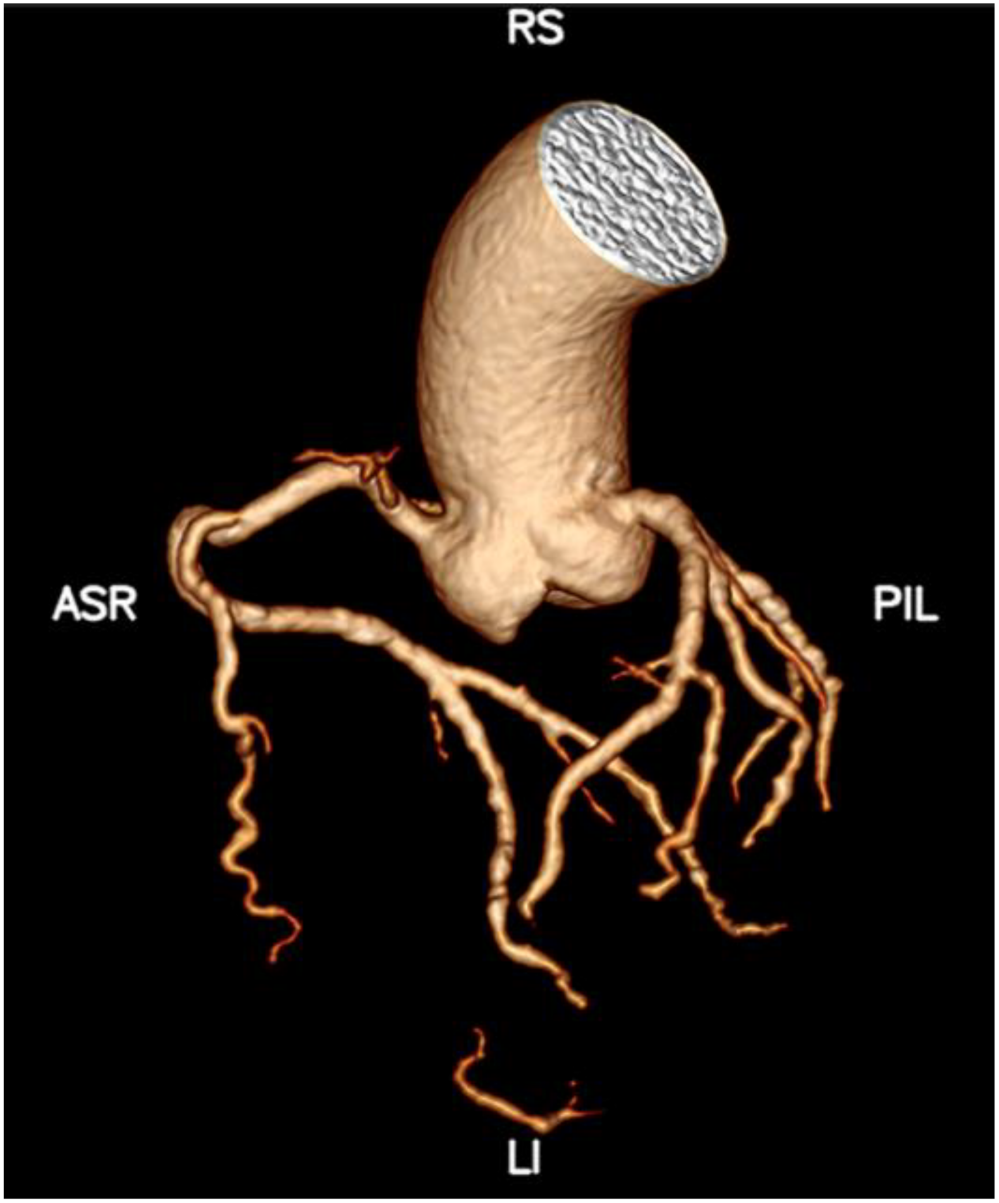
3D reconstructed coronary CTA images.

On June 29, 2025 (the third day subsequent to the second cycle of infusion), the patient experienced chest tightness without apparent causes and was unable to lie down at night. This was accompanied by marked generalized fatigue, neck soreness, and difficulty in raising the head. There were no symptoms of chest pain, palpitations, fever, cough, or expectoration. Physical examination indicated a blood pressure of 161/94 mmHg, a pulse rate of 121 beats per minute, a respiratory rate of 20 beats per minute, and an SpO_2_ of 97% (with oxygen inhalation through a nasal catheter at a rate of 3 L/min). Physical examination revealed that auscultation of both lungs was generally clear, with occasional moist rales heard. No obvious edema was observed in both lower extremities. Neurological examination revealed limited abduction of the right eye (approximately 3 mm of sclera visible) and limited adduction of the left eye (approximately 3 mm of sclera visible). The muscle strength of the proximal limbs was graded 4+, and that of the distal limbs was graded 5-. No pathological signs were detected. Laboratory tests indicated hs-cTnT at 0.425 μg/L, NT-proBNP at 396.00 pg/mL, myoglobin at 2165.00 μg/L, creatine kinase (CK) at 3866 U/L, and creatine kinase-MB (CK-MB) at 186 U/L, D-dimer 400 ng/mL, and C-reactive protein(CRP) at 26.13 mg/L. Additionally, aspartate aminotransferase (AST) was measured at 198 U/L, alanine aminotransferase (ALT) at 189 U/L, total bilirubin (TBil) at 30.10 μmol/L, and lactate dehydrogenase (LDH)at 996 U/L. Electrocardiography demonstrated sinus tachycardia, first-degree atrioventricular block, incomplete right bundle-branch block, and ST-segment depression accompanied by T-wave inversion in leads II, III, aVF, and V4 - V6 (**Figure 2**). Bedside echocardiography disclosed a left ventricular ejection fraction (LVEF) of 55%, the absence of segmental abnormalities in ventricular wall motion, mild valvular regurgitation, and diminished left ventricular diastolic function. To further determine the cause, the patient underwent an urgent coronary computed tomography angiography (CTA). The CTA results revealed coronary atherosclerosis with non-calcified plaques, and the lumen with the most severe affection had approximately 30% stenosis. There were no signs of plaque rupture, thrombosis, or occlusion (**Figure 3**). Meanwhile, no obvious abnormalities were found in the lung CT scan, and no thrombi were detected by arteriovenous ultrasound of the upper and lower limbs. Considering the above-mentioned imaging findings, the possibility of a substantial elevation in myoglobin, myocardial enzymes, and troponin caused by pulmonary embolism, acute coronary events, or peripheral vascular embolism can be basically excluded.

In conjunction with the patient’s recent medication history, significant neurologic manifestations (e.g., ocular muscle involvement, proximal limb weakness, and markedly elevated creatine kinase), and positive cardiovascular findings (e.g., troponin, myoglobin, NT-proBNP, atrioventricular block), the clinical manifestations strongly indicated immune - related myocarditis with IM3OS. Regrettably, due to the patient’s rapid deterioration, it was infeasible to complete confirmatory diagnostic procedures, including invasive or time-consuming investigations such as endomyocardial biopsy, cardiac magnetic resonance imaging, and electrophysiology. Autoantibody testing (e.g., AChR, anti-muscle-specific tyrosine kinase antibody (MuSK)) necessitates delivery and has a reporting cycle of 5 to 7 days, so it was not dispatched. Therefore, the diagnosis remains at the level of clinical suspicion and has not been definitively confirmed at the pathological or immunological level.

Upon admission, the patient discontinued adebrelimab, apatinib, and tegafur immediately. Treatment was initiated with intravenous methylprednisolone at a dose of 160 mg once daily, intravenous furosemide 10 mg once daily, intravenous isosorbide dinitrate, and intravenous magnesium isoglycyrrhizinate. On June 30, there was no improvement in the patient’s clinical symptoms. Laboratory re-evaluation revealed elevated levels of hs-cTnT at 0.350 μg/L and NT-proBNP at 520 pg/mL. By July 2, the patient experienced worsening chest tightness and dyspnea, accompanied by orthopnea and difficulty lifting the head. Auscultation revealed scattered moist rales in both lower lung fields. The dose of methylprednisolone was increased to 240 mg per day, and intravenous human immunoglobulin (10 g once daily) was initiated. Despite these interventions, symptom progression continued. On July 4, methylprednisolone was escalated to 1 g per day via intravenous infusion. Following three days of high-dose corticosteroid therapy (as of July 7), laboratory markers showed improvement: hs-cTnT decreased to 0.237 μg/L, NT-proBNP declined to 320 pg/mL, myoglobin to 200.4 μg/L, CK to 326 U/L, and creatine kinase-MB to 22 U/L. The patient reported subjective relief from chest tightness and fatigue compared to baseline.

**Figure 4:**
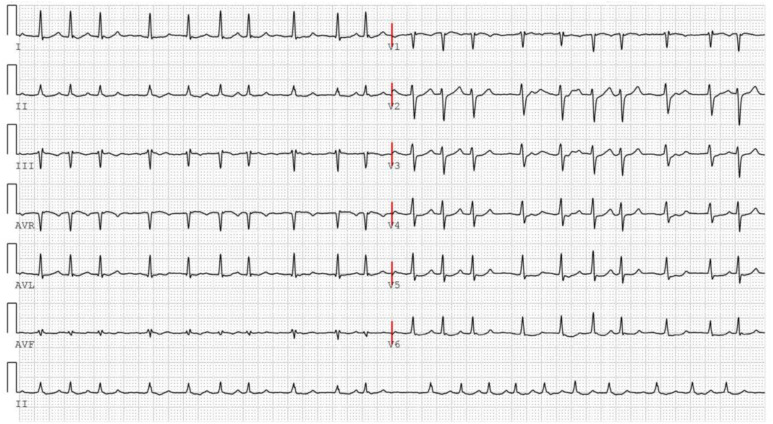
Electrocardiogram of the patient on July 8.

On the early morning of July 8, 2025, the patient’s condition underwent a sudden change. He abruptly lost consciousness, showed no response to calls, had a bilateral pupil diameter of 6mm, and the bilateral pupils were unresponsive to light. Moreover, the fluctuation of the carotid artery vanished. Cardiopulmonary resuscitation was promptly initiated. After 20 minutes of resuscitation, the patient failed to regain consciousness and remained in a coma. He was urgently transferred to the intensive care unit for continuous monitoring and advanced life support. The Glasgow Coma Scale score was recorded as 5 points (E1V1M3), and mechanical ventilation was instituted to support respiratory function. Desmopressin was administered via continuous intravenous infusion to maintain hemodynamic stability. Persistent, uncontrolled involuntary twitching of the limbs was noted during clinical observation. Physical examination demonstrates coarse breath sounds in both lungs, with moist rales audible diffusely. Moderate pitting edema is evident in both lower extremities. Mild stimulation of the limbs induces a hyperextension response, accompanied by a positive Babinski sign on the left side. Arterial blood gas analysis showed: pH 7.30, PaCO_2_ 32 mmHg, PaO_2_ 85 mmHg, HCO_3_^-^ 16 mmol/L, and lactate level of 5.8 mmol/L. Laboratory findings indicated significantly elevated cardiac biomarkers: high-sensitivity troponin T (hs-cTnT) at 0.403 μg/L, NT-proBNP at 1558 pg/mL, myoglobin at 464 μg/L, CK 766 U/L, and CK-MB 46 U/L. Electrocardiogram revealed ectopic rhythm, rapid atrial fibrillation, and ST-T segment abnormalities across multiple leads (**Figure 4**). Treatment was continued with intravenous methylprednisolone 1 g once daily in combination with intravenous immunoglobulin therapy.

On July 9, the patient remained in atrial fibrillation rhythm, with a pulse rate of 113 beats per minute. The patient was receiving ventilator - assisted breathing and was still in a comatose state. The bilateral pupils measured 5 mm, and the light reflex was slow. The Glasgow Coma Scale (GCS) score was 4 (E1V1M2). Arterial blood gas analysis revealed a pH of 7.25, PaCO_2_ of 25 mmHg, PaO_2_ of 61 mmHg, HCO_3_^-^ level of 10 mmol/L, and lactic acid level of 9.6 mmol/L. Laboratory test results indicated hs - cTnT at 0.448 μg/L, NT - proBNP at 4212 pg/mL, myoglobin at 253.5 μg/L, CK at 498 U/L, and CK-MB at 26 U/L. A chest X - ray revealed exudative changes in both lungs and pulmonary edema. Maintaining blood pressure with high doses of norepinephrine was difficult, and cardiac function continued to deteriorate. A multidisciplinary team of experts in intensive care, cardiology, neurology, and oncology concluded that there was an extremely low likelihood of meaningful neurological recovery for the patient. Following extensive discussions and verification of the patient’s previously expressed wishes to avoid prolonged suffering in the event of a poor prognosis, the family voluntarily and with informed consent decided to withdraw life-sustaining treatments. Specifically, the family requested transfer to home hospice care to allow the patient to pass away peacefully in a familiar environment, a decision consistent with their personal and cultural values. All discussions were thoroughly documented in the medical records, and formal informed consent for withdrawal of life support was obtained after institutional ethics committee review, in accordance with hospital policy and national end-of-life ethical guidelines. The patient was discharged on July 9, 2025, and passed away peacefully two days later.

## 3 Discussion

Adebrelimab is a humanized monoclonal antibody targeting PD-L1, which exerts antitumor activity by inhibiting the PD-1/PD-L1 immune checkpoint pathway. Its therapeutic efficacy has been demonstrated in multiple clinical trials, establishing it as a standard treatment option for malignancies such as extensive-stage small cell lung cancer (8). The patient first presented symptoms on the third day after the second cycle of adebrelimab infusion (24 days after the first dose), which were manifested as cardiac-related symptoms, including chest tightness, fatigue, and dyspnea, along with neuromuscular symptoms, such as ophthalmoplegia (limited abduction/adduction) and neck weakness (difficulty in head-up). Over the subsequent few days, the condition advanced rapidly, with a further deterioration of dyspnea, ultimately resulting in a fatal cardiac arrest on day 9. In terms of onset time and clinical features, this case was highly congruent with previously reported IM3OS. The patient developed symptoms 24 days after the first ICI dose, consistent with the reported median onset of 21 days, with neurological manifestations and cardiac involvement occurring concurrently (3, 4).

Regarding diagnosis, according to the 2022 ESC Cardio-Oncology guidelines, this case met the classification of “possible myocarditis”, supported by troponin elevation, new conduction abnormalities, characteristic clinical syndrome, and concurrent immune-related adverse events (9). Concurrently, the patient presented with fatigable ophthalmoplegia, neck weakness, and respiratory symptoms. Although serological and electrophysiological confirmation could not be performed due to rapid deterioration, the pattern of severe diaphragmatic weakness with only mildly reduced proximal limb strength (Grade 4+) constitutes a selective involvement pattern—a pattern that is inconsistent with the diffuse muscle weakness prerequisite for myositis-related respiratory failure but is characteristic of myasthenia gravis. Combined with the patient’s markedly elevated creatine kinase and proximal muscle weakness, these clinical features strongly suggest overlap of myositis and myasthenia gravis (10, 11).

In summary, the simultaneous occurrence of all three components—myocarditis, myasthenia gravis, and myositis—with a clear temporal relationship to ICI exposure supports the diagnosis of suspected IM3OS.

### 3.1 Etiological analysis: adebrelimab was the most probable causative factor

#### 3.1.1 Acute coronary syndrome and other ischemic events were less probable

In consideration of the existence of cardiovascular risk factors, such as multi - vessel coronary artery disease, hypertension, and diabetes, the crucial prerequisite for establishing the diagnosis of IM3OS is to clearly exclude acute coronary syndrome (ACS). ACS can be high probability excluded based on the following evidence: Firstly, serial electrocardiograms did not exhibit dynamic ST-segment elevation or evolution. The findings are more consistent with those of ICI-related myocarditis, including first-degree atrioventricular block, incomplete right bundle-branch block, and diffuse ST-T changes. Secondly, a coronary CTA conducted on the day of admission (June 29) indicated coronary atherosclerosis with non-calcified plaques, with the most severe luminal stenosis being approximately 30%, and there were no signs of plaque rupture, thrombosis, or acute occlusion. The aforementioned findings can effectively exclude ACS as the primary cause for this patient. Pulmonary embolism was reasonably excluded based on the combined evidence. The patient presented with chest tightness rather than typical sharp pleural pain. The D-dimer level was normal (400 ng/mL). No deep venous thrombosis was detected by venous ultrasound. Blood gas analysis showed only mild to moderate hypoxemia without severe hypoxemia. This combined evidence was sufficient to exclude clinically significant pulmonary embolism, even though CT pulmonary angiography was not performed.

#### 3.1.2 Viral myocarditis was less probable

Viral myocarditis is a prevalent cause of myocarditis and should be incorporated into the differential diagnosis. Common pathogenic viruses encompass Coxsackievirus B (30% - 50%), echovirus, adenovirus, Epstein-Barr virus, cytomegalovirus, and influenza virus. Nevertheless, the likelihood of viral myocarditis in this case is extremely low (12). Firstly, the prodromal symptoms typical of viral myocarditis (fever, sore throat, myalgia, and diarrhea) were absent. Secondly, the patient presented with a triad of involvement of the heart, skeletal muscle, and ocular muscles (myocarditis, myositis, and ophthalmoplegia)-a highly characteristic finding of ICI-related IM3OS that is not typical of viral myocarditis. Hence, this is the rationale for not conducting viral serology as an urgent test upon admission.

#### 3.1.3 Transcatheter arterial chemoembolization (TACE)-associated cardiac toxicity was less probable

On May 22, 2025, the patient underwent TACE. During this procedure, an infusion of 2 mg of raltitrexed and 100 mg of oxaliplatin was carried out, followed by embolization using 30 mg of pirarubicin and 10 ml of iodized oil. The probability of a direct causal link between this treatment regimen and the subsequent suspected IM3OS on June 29 is extremely low. TACE was administered locally, and the dosage was notably lower than that of systemic chemotherapy, leading to limited systemic exposure. A single dose of 30 mg of pirarubicin (approximately 17.6 mg/m²) was well below the anthracycline cardiotoxicity threshold (>250 mg/m²) (13). Raltitrexed, which serves as a standard alternative regimen for patients unsuitable for fluorouracil chemotherapy because of cardiovascular toxicity, has a low risk of cardiovascular events, mainly presenting as angina pectoris and arrhythmia (14). Oxaliplatin cardiotoxicity is rare, mainly manifested as QT interval prolongation and coronary spasm (15). All three drugs have an extremely low likelihood of causing myocarditis. Therefore, combined with the dose of administration and the type of adverse reactions, the possibility of TACE regimen directly causing IM3OS is ruled out with a high probability. Nevertheless, the inflammatory response subsequent to TACE may induce a susceptible state of the myocardium, creating a potential setting for subsequent ICI immunotoxicity. (The formal WHO-UMC causality assessment for these TACE agents is presented in Section 1.4 below).

#### 3.1.4 Drug causality assessment

To elucidate the causal relationship between the drugs and IM3OS, a systematic analysis was carried out using the WHO-UMC causality evaluation system (16). The evaluation system was assessed from six dimensions: temporal relevance, pharmacological rationality, de-challenge response, re-challenge response, exclusion of other causes, and literature evidence.

For TACE agents (raltitrexed, oxaliplatin, pirarubicin): Although a temporal relationship exists, these agents lack pharmacological plausibility for inducing IM3OS, as they do not activate T cells or disrupt immune tolerance. There was no de-challenge response, no re-challenge, alternative causes cannot be completely excluded, and there is no literature evidence supporting IM3OS associated with these drugs. According to the WHO-UMC system, these agents are each rated as “unlikely”.

For apatinib and tegafur: Apatinib is a VEGFR-2 tyrosine kinase inhibitor. Its adverse reactions are primarily attributable to vascular-targeted toxicity (hypertension, proteinuria, hand-foot skin reaction, myocardial ischemia) and it lacks an immune activation mechanism (9, 17, 18). Tegafur is a prodrug of 5-fluorouracil; its cardiotoxicity is mediated through direct cytotoxicity (coronary spasm, arrhythmia, QT prolongation) rather than immune activation (19). Both drugs lack pharmacological justification for causing IM3OS, have no support from relevant literature, and there is a more probable primary causative factor. According to the WHO-UMC system, both are rated as “unlikely”.

For adebrelimab: The temporal relationship is clear (symptom onset 24 days after the first dose, consistent with the reported median onset of 21 days). The pharmacological mechanism is well-established (PD-L1 blockade leading to T-cell hyperactivation and cross-reactive infiltration of cardiac, skeletal muscle, and neuromuscular junction tissues). The literature supports its association with IM3OS. Under the strictest application of the WHO-UMC criteria, adebrelimab is therefore rated as “possible”.

### 3.2 Reflections on therapy: from “escalation” to “initial reinforcement”

The treatment process and final outcome of this case offer profound reflections on the management strategy of IM3OS. Although the clinical team promptly ceased the previous treatment and initiated glucocorticoid therapy in line with the guidelines, the approach of dose escalation (from 160 mg to 240 mg per day, with a 1 - g pulse) might not have been effective in containing the rapidly progressing immune storm. The factors influencing the decision at that time were as follows: uncertainty in diagnosis upon admission (acute coronary syndrome, infection, etc., needed to be excluded); cognitive biases [misinterpreting an LVEF of 55% as an indicator of cardiac safety while overlooking electrical instability as a primary cause of death in ICI myocarditis (20); misclassifying a moderate troponin elevation of 0.425 μg/L as “moderate disease,” even though studies have demonstrated fatal events associated with mild biomarker elevations (21)]; and the risk of hormonal complications due to comorbidities such as advanced age, diabetes, and hypertension.

Reasons for not using Plasma exchange (TPE): It is important to note that despite TPE being recommended as one of the first-line treatments for severe IM3OS, it was not employed in this particular case. From July 4th to 7th, the patient exhibited transient clinical improvement, as evidenced by a decrease in hs-cTnT, which indicated a partial response to high-dose hormone therapy. This improvement created a false sense of security, leading to the perception that TPE was not urgently required. On July 8th, the patient suffered a sudden cardiac arrest, following which the clinical focus shifted towards life-support measures. In our institution, TPE necessitates 12–24 hours of multidisciplinary coordination. However, this time window no longer existed after the patient’s condition continued to deteriorate. After a comprehensive discussion with the patient’s family, further invasive intervention was ultimately abandoned.

In light of the high mortality rate of IM3OS, the traditional “step-by-step” or “reactive” treatment escalation approach frequently fails to seize the optimal treatment window because it lags behind the progression of the disease. Consequently, there is an urgent requirement for a shift in the clinical strategy towards an “intensified initial immunosuppression” paradigm. For patients with a high degree of suspicion or confirmed severe IM3OS, the most potent combination immunosuppressive regimen should be promptly initiated within 24–48 hours upon recognition. The core components of this regimen encompass: High - dose glucocorticoid pulse therapy (e.g., methylprednisolone 500–1000 mg/day) combined with intravenous immunoglobulin (IVIG, 2 g/kg) and/or therapeutic plasma exchange (22–24). This regimen is formulated to rapidly eliminate pathogenic antibodies, neutralize inflammatory factors, and comprehensively suppress the function of effector T-cells through multiple mechanisms. It is essential to establish a rapid multidisciplinary team response mechanism involving oncology, cardiology, neurology, and critical care medicine, and to offer comprehensive organ support treatment, including respiratory support (early tracheal intubation if necessary), continuous hemodynamic and electrocardiographic monitoring, and active management of arrhythmias.

### 3.3 Exacerbation mechanisms: electrical storm and the “second hit”

On July 7, the patient’s troponin level briefly decreased, indicating partial control of inflammation. However, on July 8, the patient experienced sudden cardiac arrest. This paradoxical phenomenon can be elucidated by the following mechanisms: Firstly, cardiac biomarkers have inherent limitations, and major adverse cardiovascular events can still transpire even when CK and cTnI return to normal (21). Secondly, the myocardium may still remain electrically unstable. The fragile matrix can trigger an electrical storm, and sustained ventricular tachycardia causes secondary myocardial injury by augmenting oxygen consumption and reducing coronary perfusion, resulting in a rebound of troponin (20, 25). Thirdly, the electrical storm further exacerbates immune disorders and respiratory failure. Arrhythmias lead to a decrease in cardiac output and hypoperfusion of the diaphragm and intercostal muscles, which exacerbate pre-existing respiratory muscle weakness. Ischemia-reperfusion injury after cardiac arrest releases DAMPs (such as HMGB1 and mitochondrial DNA), activates innate immunity via the TLR4 and NLRP3 pathways, and releases pro-inflammatory cytokines such as interleukin-1β (IL-1β), interleukin-6 (IL-6), and tumor necrosis factor-α (TNF-α). These cytokines, on the one hand, aggravate myocardial injury and increase alveolar capillary permeability, leading to non - cardiogenic pulmonary edema (26, 27). These mechanisms collectively contribute to the rapid progression to respiratory failure despite the improvement in biomarkers.

### 3.4 Diagnostic limitations and their clinical implications

Ideal diagnostic tests for IM3OS should encompass cardiac magnetic resonance, endomyocardial biopsy, autoantibody detection (AChR, MuSK, anti-striated muscle antibody), electrophysiological studies, virus screening, inflammatory markers (CRP, erythrocyte sedimentation rate (ESR), IL-6, aldolase), and cardiac biomarkers (3, 9, 24). Nevertheless, despite this ideal framework, several practical issues have hindered the completion of these examinations. Firstly, the fulminant clinical course, which lasted only 9 days from admission to cardiac arrest, prioritized therapeutic intervention over time-consuming investigations. Secondly, hemodynamic instability and orthopnea prevent the conduct of cardiac MRI, as it necessitates extended periods of lying flat and breath-holding. Thirdly, electrophysiological studies were not carried out. Prior to the cardiac arrest, the patient was unable to tolerate lying flat due to orthopnea (a process that takes 30–60 minutes). The disturbance of consciousness after cardiac arrest (GCS 4 - 5) precludes any examination requiring the patient’s cooperation. Fourthly, skeletal-muscle biopsy and endomyocardial biopsy could not be conducted because of procedural risks and patient instability. Fifthly, an autopsy could not be performed, as the family declined consent for personal and cultural reasons.

Given that the detection of autoantibodies (AChR, MuSK, anti-striated muscle antibody) is not a routine procedure in our hospital, the samples need to be sent to a third-party reference laboratory. The delivery process must strictly adhere to the hospital’s standards, and the entire process takes 5–7 days. More significantly, the diagnosis and treatment decisions for IM3OS are primarily based on clinical manifestations and do not rely on antibody results. According to a systematic review conducted by Ghosh et al., the prevalence of striated muscle antibodies in ICI-related IM3OS was approximately 49%, that of AChR antibodies was approximately 40%, and that of myositis-related antibodies was approximately 27% (28). This indicates that a substantial proportion (around 40 – 50%) of patients are seronegative, a negative result does not exclude the diagnosis, and a positive result does not alter the immediate treatment strategy. The standard of care for suspected severe IM3OS is the immediate initiation of high-dose immunosuppression (corticosteroids ± IVIG ± TPE), regardless of the antibody status. Therefore, considering the timeliness of testing, clinical practicality, and the independence of antibody results for treatment decisions comprehensively, autoantibody testing was not initiated upon admission.

This case indicates that IM3OS is a clinical diagnosis that demands urgent management, with a narrow therapeutic window. Clinicians should identify “red alert” signals at an early stage. Once the diagnosis is strongly suspected, it is essential to actively conduct confirmatory tests and initiate high-dose immunosuppressive therapy immediately, even for elderly patients with comorbidities. These limitations genuinely reflect the clinical challenges in managing this rapidly fatal syndrome.

## 4 Conclusion

In conclusion, this patient developed fulminant immune checkpoint inhibitor- associated myocarditis accompanied by suspected IM3OS within the typical window period following the combined regimen of adebrelimab, apatinib, and tegafur. The clinical course of this patient was highly consistent with the immunotoxicity mechanism. This case serves as a most severe warning for clinical practice: when employing such potent combination regimens, a meticulous pretreatment baseline risk assessment must be conducted, very early and active monitoring of symptoms and biomarkers must be carried out, and a plan should be formulated to ensure that once severe immunotoxicity is detected, the most effective combination intervention can be initiated as rapidly as possible. Only in this way can we firmly uphold the bottom line of patient safety while pursuing anti - tumor efficacy.

## Data Availability

The original contributions presented in the study are included in the article/supplementary material. Further inquiries can be directed to the corresponding author.
